# Personal sense of coherence and posttraumatic stress symptoms among internally displaced women exposed to the Anglophone Crisis in Cameroon

**DOI:** 10.3389/fgwh.2026.1894160

**Published:** 2026-09-03

**Authors:** Edwige Emilie Kouamen Tatto Mouandjo, Anne Wandjiru Mbwayo, Anne Muthoni Mathai, Jean-Baptiste Fotso Djemo, Houdou Seyni, Thomas Schnell, Julia Fischer, Roland Weierstall-Pust, Gesa Pust

**Affiliations:** 1 MONUSCO—United Nations Organization Stabilization Mission in the DR Congo, Kinshasa, Democratic Republic of Congo; 2 University of Nairobi, Nairobi, Kenya; 3 Université des Montagnes, Bangangté, Cameroon; 4 International Medical Corps, Kinshasa, Democratic Republic of Congo; 5 MSH Medical School Hamburg, University of Applied Sciences and Medical University, Hamburg, Germany

**Keywords:** anglophone crisis, Cameroon, community coherence, internally displaced persons, posttraumatic stress disorder, resilience, sense of coherence, trauma load

## Abstract

Women and girls affected by armed conflict and forced displacement are exposed to a high burden of potentially traumatic events, gendered insecurity, disrupted social networks, and barriers to mental health care. Evidence on protective psychological resources among internally displaced women in Central Africa remains limited. This study examined trauma load, personal sense of coherence (SoC), and sense of community coherence (SoCC) as predictors of posttraumatic stress disorder (PTSD) symptom severity among women internally displaced by the Anglophone Crisis in Cameroon. A sample of 133 female internally displaced persons (IDPs), aged 18 to 65 years, was recruited from two humanitarian organizations. Trauma exposure was assessed using the Life Events Checklist for DSM-5 and a crisis-specific trauma checklist; PTSD symptom severity was measured with the PTSD Checklist for DSM-5; and SoC and SoCC were assessed with validated self-report scales. Among six trauma-load indicators, self-experienced traumatic event types related to the Anglophone Crisis were the only trauma-related predictor retained in the final regression model. The final model included self-experienced Anglophone Crisis-related trauma (*β* = .23, *p* = .009) and personal SoC (*β* = −.18, *p* = .035), *F*(2, 130) = 7.41, *p* < .001, adjusted *R*^2^ = .09. Using the PCL-5 screening cut-off of 31, 127 of 133 women (95.5%) met the threshold for probable PTSD. The findings do not establish causal mediation, but they support the interpretation that individual comprehensibility, manageability, and meaningfulness are clinically relevant resilience-related resources for displaced women. Interventions for female IDPs should combine trauma-focused care with strategies that strengthen individual coping and meaning-making while acknowledging that displacement can disrupt community-level protection.

## Introduction

Armed conflict and forced displacement create profound risks for mental health. Women and girls frequently experience these risks within gendered social, economic, and relational conditions that can amplify both exposure to violence and barriers to recovery. In humanitarian settings, psychological distress rarely reflects a single traumatic event alone. It often emerges from cumulative threat, loss, forced relocation, separation from social networks, caregiving strain, poverty, and reduced access to health and psychosocial support. Recent reviews of women's mental health in fragile and humanitarian settings emphasize that women exposed to conflict and displacement show a high burden of posttraumatic stress, anxiety, depression, and psychological distress, while services often remain under-resourced and insufficiently tailored to women's needs (1). For internally displaced persons (IDPs), these risks may be especially pronounced because displacement occurs within national borders and may not be accompanied by the same legal and institutional support structures available to refugees.

The present study focuses on female IDPs affected by the Anglophone Crisis in Cameroon. The conflict in the North-West and South-West regions is rooted in colonial and post-colonial histories, including the political and institutional marginalization of Anglophone communities after reunification with the Francophone-majority state (2, 3). Since the escalation of violence in 2017, the conflict has involved confrontations between state forces and separatist groups, with severe consequences for civilians. Reported human rights violations have included killings, abductions, sexual assaults, torture, destruction of homes, loss of livelihood, and forced displacement. These experiences are not only threats to physical safety; they also disrupt social roles, family structures, education, health care, and expectations of the future. For displaced women, such disruption may be compounded by responsibility for children and family members, economic dependence, social stigma, and the threat of gender-based violence.

PTSD represents one of the most studied psychological consequences of traumatic exposure. The disorder can develop after exposure to actual or threatened death, serious injury, or sexual violence and is characterized by symptoms such as intrusive memories, avoidance, negative alterations in cognition and mood, and hyperarousal (4). In conflict-affected populations, PTSD symptoms often co-occur with depression, anxiety, somatic complaints, grief, and functional impairment. A recent systematic review and meta-analysis among IDPs in Africa reported a high pooled prevalence of PTSD and identified female gender, number of traumatic events, repeated displacement, illness without medical care, marital status, unemployment, and depression as relevant correlates (5). These findings underline the need for research that considers both cumulative exposure and protective resources in African displacement contexts.

A central and robust finding in trauma research is the dose-response association between cumulative traumatic exposure and PTSD symptom severity. This relationship has often been conceptualized as a “building block effect,” meaning that the probability and severity of PTSD symptoms increase as the number of different traumatic event types increases (6–8). The building block model is highly relevant in contexts of war and forced displacement, where individuals may be exposed to repeated and qualitatively different threats over months or years. Mechanistically, cumulative trauma may alter biological stress systems, increase hyperarousal and vigilance, consolidate threat-related memories, and shape maladaptive appraisals of the self, others, and the world (9–11).

However, trauma exposure alone does not fully explain who develops severe PTSD symptoms and who remains comparatively resilient. Individuals with similar trauma loads may differ markedly in symptom severity, functional impairment, and recovery trajectories. These differences point to the importance of psychological and social resources that influence how traumatic experiences are appraised, organized, and integrated into autobiographical memory and identity (12, 13, 39–41). In forced displacement contexts, it is also important to distinguish between different modes of exposure. DSM-5 Criterion A includes direct experience, witnessing, learning about traumatic events affecting close others, and repeated exposure as part of professional duties (4). Yet these categories may not contribute equally to PTSD symptom severity in every setting. In contexts where witnessing violence and hearing about atrocities are common, personally experienced events may carry particular salience and differentiating power.

Women's trauma exposure also has specific characteristics that warrant focused study. A systematic review of refugees, IDPs, and asylum seekers found sex differences in both trauma exposure and PTSD symptom presentation, with displaced women reporting higher rates of sexual violence and showing different symptom patterns compared with men (14). These findings do not imply that women constitute a homogeneous group; rather, they emphasize that trauma and recovery are shaped by gendered risks, social expectations, and access to resources. Research on women's mental health in humanitarian settings must therefore examine not only trauma load but also resilience resources that may be clinically and culturally meaningful.

Conflict-related trauma and PTSD symptoms in these samples may be related in different ways: Conflict and displacement can expose women to sexual and gender-based violence, coercion, reproductive health threats, stigma, economic dependence, or caregiving responsibilities under conditions of insecurity. These stressors may intensify perceptions of threat, feelings of shame and guilt, loss of control, and social isolation, while also limiting access to protection and psychosocial support (14, 15). A women-focused analysis is therefore not only a matter of sample description, but also a way to examine trauma and resilience in a social context shaped by gendered exposure, resources, and barriers to recovery.

Sense of coherence (SoC) offers one such framework. Originating in Antonovsky's salutogenic theory, SoC refers to a global orientation through which individuals perceive life as comprehensible, manageable, and meaningful (16, 17). Comprehensibility reflects the extent to which internal and external experiences are perceived as structured and understandable; manageability refers to the perception that resources are available to meet demands; and meaningfulness captures the sense that challenges are worthy of engagement. In the context of trauma, SoC may influence whether survivors can organize overwhelming experiences within a coherent narrative, identify coping resources, and maintain a sense of purpose despite loss and uncertainty.

Empirical evidence supports the relevance of SoC for trauma-related outcomes. Meta-analytic work has shown a substantial inverse association between SoC and posttraumatic stress reactions, indicating that higher SoC is consistently related to lower PTSD symptom severity (18, 19). Studies in high-risk occupational and survivor populations also suggest that SoC may function as a resilience-related resource in the face of repeated stress and threat (20, 21). In a displacement setting, personal SoC may be especially relevant because the individual must repeatedly adapt to unpredictable living conditions, loss of familiar routines, and constrained access to institutional support.

At the same time, resilience is not exclusively individual. Social support, collective efficacy, perceived safety, and a sense of belonging can shape mental health after collective violence (22, 23). The concept of sense of community coherence (SoCC) extends the salutogenic perspective to the community level. SoCC refers to the perception that one's community is comprehensible, manageable, and meaningful as a collective environment (24, 25). A coherent community may offer shared narratives, mutual support, predictable norms, and practical resources that buffer the effects of trauma. In adolescents and populations exposed to political violence, community-level resources have been linked to coping and psychological outcomes (26, 27).

Nevertheless, the protective role of SoCC may be altered by internal displacement. Displacement can separate people from their community of origin, place them in unfamiliar host communities, and weaken the continuity of social identity and support. For women who have fled conflict zones, “community” may no longer refer to a stable and trusted network. It may instead be fragmented across the place of origin, current settlement, humanitarian organizations, religious networks, and informal support structures. Under such conditions, community coherence may be difficult to access even if collective resources remain valued. This makes it important to examine SoC and SoCC simultaneously rather than assuming that community-level coherence automatically buffers PTSD symptoms in displaced populations.

The present study was designed to examine these issues among female IDPs affected by the Anglophone Crisis in Cameroon. Specifically, we assessed six trauma-load indicators that distinguished between non-war-related and Anglophone Crisis-related event types and between self-experienced, witnessed, and learned-about exposure. We then examined whether personal SoC and SoCC were associated with PTSD symptom severity and whether either construct moderated the relationship between trauma load and PTSD symptoms. Based on prior evidence, we expected greater trauma load to be associated with higher PTSD symptom severity and higher personal SoC to be associated with lower symptom severity. Because the role of SoCC in displaced women's PTSD remains less well established, analyses involving SoCC were considered exploratory. The novelty of the study lies in examining these elements simultaneously in an underrepresented sample of female IDPs affected by the Anglophone Crisis: a differentiated trauma-load model, personal SoC, SoCC, and their independent and moderating associations with PTSD symptom severity.

## Materials and methods

### Study design and setting

This cross-sectional study examined trauma exposure, personal and community coherence, and PTSD symptom severity among women internally displaced by the Anglophone Crisis in Cameroon. Participants were recruited through two humanitarian organizations that provided contact with IDPs and supported local coordination: Trauma Centre Cameroon (TCC) and HaRO (Hope and Rehabilitation Organisation). These organizations were selected because they were directly engaged with displaced populations from the North-West and South-West regions and maintained registration lists that allowed systematic recruitment. The study was designed as an observational field study in a humanitarian context, with procedures adapted to protect participants' safety, autonomy, and confidentiality.

Data collection took place in Yaoundé among women who had left the conflict-affected regions and had spent at least three months outside their places of origin. The focus on women was intentional and aligned with the study aim of examining women's mental health and resilience after conflict-related internal displacement. Because the study was conducted in a low-resource and conflict-affected setting, the assessment used brief self-report instruments and a trauma checklist adapted to the local crisis context. This approach aimed to balance scientific rigor with feasibility and participant burden.

### Participants and recruitment

Participants were recruited from IDP registration lists maintained by TCC and HaRO. At the time of assessment, the organizations had approximately 105 and 397 registered IDPs, respectively. An *a priori* sample size calculation using G*Power 3.1.9.7 (28) indicated a minimum required sample of 107 participants. To reflect the size of the two organizational populations, participants were drawn in proportion to the number of registered IDPs in each organization. Every fourth person on the respective registration lists was approached for participation. When a selected person did not meet inclusion criteria or declined participation, the next eligible person was selected according to the same systematic procedure until the planned sample size was reached.

Eligibility criteria were defined before recruitment. Participants had to be female, at least 18 years old on the day of assessment, internally displaced as a result of the crisis in the North-West or South-West regions, and originally from one of these regions. Persons with acute and severe health impairments that would have made participation unsafe or impossible were not included. Individuals who appeared to need further psychological assistance could be referred to the psychological team of Trauma Centre Cameroon. Participation was voluntary and anonymous, and refusal to participate had no consequences for the support received from the organizations.

The final sample consisted of 133 female IDPs between 18 and 65 years of age (*M* = 34 years, SD = 11). Participants had spent a minimum of three months in Yaoundé since leaving the conflict zones. Demographic variables included age, marital status, number of children, educational level, region of origin, and subjective family economic status. The assessment of family economic status was based on participants' self-ratings. No statistically significant differences in socio-demographic variables were observed between women from the North-West and South-West regions.

Because recruitment was limited to women registered with or reachable through two humanitarian organizations, the sampling frame may have introduced selection bias. Women who were not registered, who had limited access to humanitarian services, who lived outside Yaoundé, who remained in conflict-affected areas, or who had returned to their places of origin may be underrepresented. Accordingly, the sample should be understood as a systematically recruited service-connected IDP sample rather than a representative sample of all internally displaced women affected by the Anglophone Crisis.

### Ethical considerations and procedure

Ethical approval was obtained from the KNH-UON Ethics Board of the University of Nairobi, the University of Douala Board of Ethics, and the responsible leadership of HaRO. All participants received information about the study aims, procedures, voluntary nature of participation, anonymity, and the possibility of discontinuing participation at any time. Written informed consent was required before the assessment. Participants received 1,500 CFA francs (approximately 2.58 USD) to cover transport costs from their neighborhood to the study site and back.

Participants were contacted through the two organizations and invited to attend an assessment appointment at a time that was convenient for them. Upon arrival, participants were welcomed and directed to a safe and private setting for data collection. The study aims and procedures were explained again in accessible language. After written informed consent was obtained, participants completed the questionnaires. A mental health professional from the study team was available to respond to questions, clarify procedures without influencing answers, and assist in the event of distress. The assessment was designed to minimize participant burden while still capturing the main constructs relevant to trauma exposure, PTSD symptoms, and coherence resources.

### Measures

#### Trauma load

Trauma load was operationalized as the number of different traumatic event types reported by each participant. Counting event types rather than frequencies is a common approach in research on war-affected and displaced populations and is consistent with previous studies that have used trauma-load scores to examine dose-response associations with PTSD symptoms (29, 30). Two trauma checklists were administered. The first was the Life Events Checklist for DSM-5 (LEC-5), which assesses exposure to potentially traumatic events (31). To reduce artificial overlap with the crisis-specific checklist, war-zone-related LEC-5 items were removed, resulting in a list of ten non-war-related potentially traumatic event types.

The second checklist was a 20-item list of traumatic event types specific to the Anglophone Crisis. This checklist was developed by Cameroonian mental health experts working with war-affected IDPs to reflect major event types experienced in the conflict context. It was intended to capture the local profile of conflict-related victimization, including events such as loss of loved ones, detention or threat in a war zone, killings, kidnapping, and other crisis-related violations. For both checklists, exposure was categorized according to DSM-5 Criterion A distinctions: self-experienced events, witnessed events, learned-about events, and events experienced as part of professional duties (4). The professional-duty category was not included in statistical analyses because only one participant endorsed one event in this category across all trauma categories. The item pool was generated from field-based clinical experience, related publications and contextual knowledge of Cameroonian mental health professionals working with war-affected IDPs. All items were reviewed for context-specific relevance, clarity, and coverage of salient crisis-related event types. The checklist has not yet undergone formal psychometric validation as a standalone instrument; therefore, it was used as a context-specific event-type inventory to quantify trauma load, not as a diagnostic measure.

The resulting six trauma-load scores were: LEC-5 non-war self-experienced events, LEC-5 non-war witnessed events, LEC-5 non-war learned-about events, Anglophone Crisis self-experienced events, Anglophone Crisis witnessed events, and Anglophone Crisis learned-about events. This differentiation was important because not all forms of exposure may have equal predictive value for PTSD symptom severity in a protracted conflict setting. In particular, self-experienced events may represent the most personally salient forms of exposure, whereas witnessed and learned-about events may be widespread within communities affected by violence.

#### PTSD symptom severity

PTSD symptom severity was measured with the PTSD Checklist for DSM-5 (PCL-5). The PCL-5 is a 20-item self-report measure based on DSM-5 PTSD symptom criteria and has shown good psychometric properties in previous research (32). Participants rated each symptom on a 5-point scale from 0 (“not at all”) to 4 (“extremely”), yielding a total score ranging from 0 to 80. Higher scores indicate greater PTSD symptom severity. In the present manuscript, the outcome is consistently referred to as PCL-5 symptom severity. This replaces the earlier inconsistent wording in which the outcome had also been labeled PSS-I. A cut-off range of approximately 31–33 has often been used to indicate probable PTSD in screening contexts, although cut-offs are not substitutes for clinical diagnosis (33). The PCL-5 has shown excellent internal consistency (*α* = .94) (34).

#### Personal sense of coherence

Personal SoC was assessed with the 13-item short form of the Sense of Coherence Scale (SOC-13). The SOC-13 measures the three core components of Antonovsky's salutogenic model: comprehensibility, manageability, and meaningfulness (17, 35). Items are rated on a 7-point scale. Higher total scores indicate a stronger sense of coherence. In this study, personal SoC was conceptualized as an individual resilience-related resource that may help survivors organize, interpret, and cope with stressful and traumatic experiences. The SOC-13 has demonstrated acceptable to excellent reliability across diverse settings, including African samples (*α* = .70–.92) (36).

#### Sense of community coherence

SoCC was assessed with the Sense of Community Coherence questionnaire, a 7-item self-report scale designed to capture the extent to which individuals perceive their community as comprehensible, manageable, and meaningful (24, 25). Items are rated on a Likert scale from 0 to 6. Higher scores indicate stronger perceived community coherence. In this study, SoCC was included because community-level resources may be particularly relevant in collective violence and displacement. At the same time, displacement may weaken the practical and emotional accessibility of community coherence, making its role an empirical question rather than an assumption. The Sense of Community Coherence Questionnaire has shown good internal consistency in prior research (*α* = .82–.85) (37).

### Statistical analysis

The statistical analyses followed a planned stepwise approach. First, descriptive analyses were used to characterize the sample and to identify the most frequently endorsed traumatic event types within each of the six trauma-load categories. The top three event types in each category were reported to describe the qualitative profile of trauma exposure and to distinguish non-war-related from Anglophone Crisis-related exposure. Second, zero-order correlations were calculated among PCL-5 symptom severity, personal SoC, SoCC, and the six trauma-load scores. These correlations were used to provide an initial overview of bivariate associations and to evaluate whether the observed pattern was consistent with the planned regression analyses.

Third, a multiple linear regression model was used to predict PCL-5 symptom severity from the six trauma-load scores. Non-significant predictors were removed using a backward exclusion procedure. This procedure was chosen to identify which trauma-load category retained predictive value when the different exposure categories were considered simultaneously. Multicollinearity was evaluated using variance inflation factors (VIFs). The trauma-load score retained in the final model was then used in the subsequent model including personal and community coherence variables. The backward procedure was used as a pragmatic data-reduction strategy to identify the exposure category with independent predictive value while limiting the number of predictors in relation to the modest sample size and the correlations among trauma-load indicators. However, stepwise procedures can be sample-dependent and may increase the risk of model-selection bias; therefore, the resulting models are interpreted as parsimonious exploratory models rather than definitive variable-selection tests.

Fourth, the role of personal SoC and SoCC was examined in a regression model that included self-experienced Anglophone Crisis-related trauma load, personal SoC, SoCC, and the interaction terms between trauma load and each coherence variable. Before calculating interaction terms, variables were mean-centered to reduce non-essential multicollinearity. Non-significant predictors were again removed using backward exclusion. The interaction terms tested whether personal SoC or SoCC moderated the association between trauma load and PCL-5 symptom severity.

The original manuscript framed this analysis in terms of mediation and moderation. After rechecking the statistical pattern, the interpretation was refined. Because both self-experienced Anglophone Crisis-related trauma load and personal SoC remained significant predictors in the final model, and because no formal indirect effect was estimated, the present version does not claim full mediation. Instead, the results are interpreted as showing that personal SoC is independently and inversely associated with PTSD symptom severity after adjustment for trauma load. The observed zero-order correlations are compatible with a possible indirect or partial explanatory pathway, but causal mediation cannot be concluded from the present cross-sectional regression analyses alone. All analyses were conducted using Stata and R for Windows. The level of statistical significance was set to alpha = .05, and effect sizes and model fit indices were reported where available. Regression tables were expanded to include 95% confidence intervals for the regression coefficients.

## Results

### Sample characteristics

Table 1 presents the demographic characteristics of the sample. The final sample included 133 female IDPs, with a median age of 34 years. Most participants were between 30 and 44 years of age, followed by the 18–29 age group. Nearly half of the women were single, approximately one third were married or cohabiting, and the remaining participants were widowed, divorced, or separated. Most participants had children, with a median of three children. Educational attainment was distributed across primary, secondary, and tertiary education, with secondary education representing the largest group. Family economic status was predominantly rated as much less than average or less than average, indicating substantial economic hardship in the sample.

**Table 1:** Demographic characteristics of the sample.

Variable	*N*	Overall, *N* = 133	North-West, *N* = 55	South-West, *N* = 78	Test statistic
Age category (years)	133				*χ*2 = 0.242, *p* = .886
18–29		47 (35%)	20 (36%)	27 (35%)	
30–44		66 (50%)	26 (47%)	40 (51%)	
45+		20 (15%)	9 (16%)	11 (14%)	
Age in years	133	34 (27, 41)	33 (27, 41)	35 (27, 41)	
Marital status	133				χ2 = 2.393, *p* = .302
Single		61 (46%)	21 (38%)	40 (51%)	
Married/cohabiting		47 (35%)	23 (42%)	24 (31%)	
Widowed/divorced/separated		25 (19%)	11 (20%)	14 (18%)	
Number of children	133				χ2 = 1.654, *p* = .647
None		19 (14%)	7 (13%)	12 (15%)	
1–2 children		35 (26%)	16 (29%)	19 (24%)	
3–4 children		38 (29%)	13 (24%)	25 (32%)	
5+ children		41 (31%)	19 (35%)	22 (28%)	
Number of children, median (IQR)	133	3.00 (1.00, 5.00)	3.00 (1.50, 5.00)	3.00 (1.00, 5.00)	
Level of education	132				χ2 = 0.636, *p* = .728
Primary		31 (23%)	14 (26%)	17 (22%)	
Secondary		69 (52%)	26 (48%)	43 (55%)	
Tertiary		32 (24%)	14 (26%)	18 (23%)	
Missing		1	1	0	
Family economic status	133				χ2 = 2.853, *p* = .240
Much less than average		61 (46%)	30 (55%)	31 (40%)	
Less than average		58 (44%)	20 (36%)	38 (49%)	
Average		14 (11%)	5 (9.1%)	9 (12%)	

Note. Values are *n* (%) or median (IQR), unless otherwise indicated. Test statistics are Pearson's chi-square test, Wilcoxon rank-sum test, or Fisher's exact test as appropriate.

Participants originated from both major conflict-affected Anglophone regions. Fifty-five women were from the North-West region and 78 from the South-West region. There were no statistically significant differences between these regional groups in age category, marital status, number of children, level of education, or subjective family economic status. This pattern suggests that the two regional subgroups were broadly comparable with respect to the measured socio-demographic characteristics, allowing the full sample to be analyzed together in the main models.

### Traumatic event types across exposure categories

**Figure 1:**
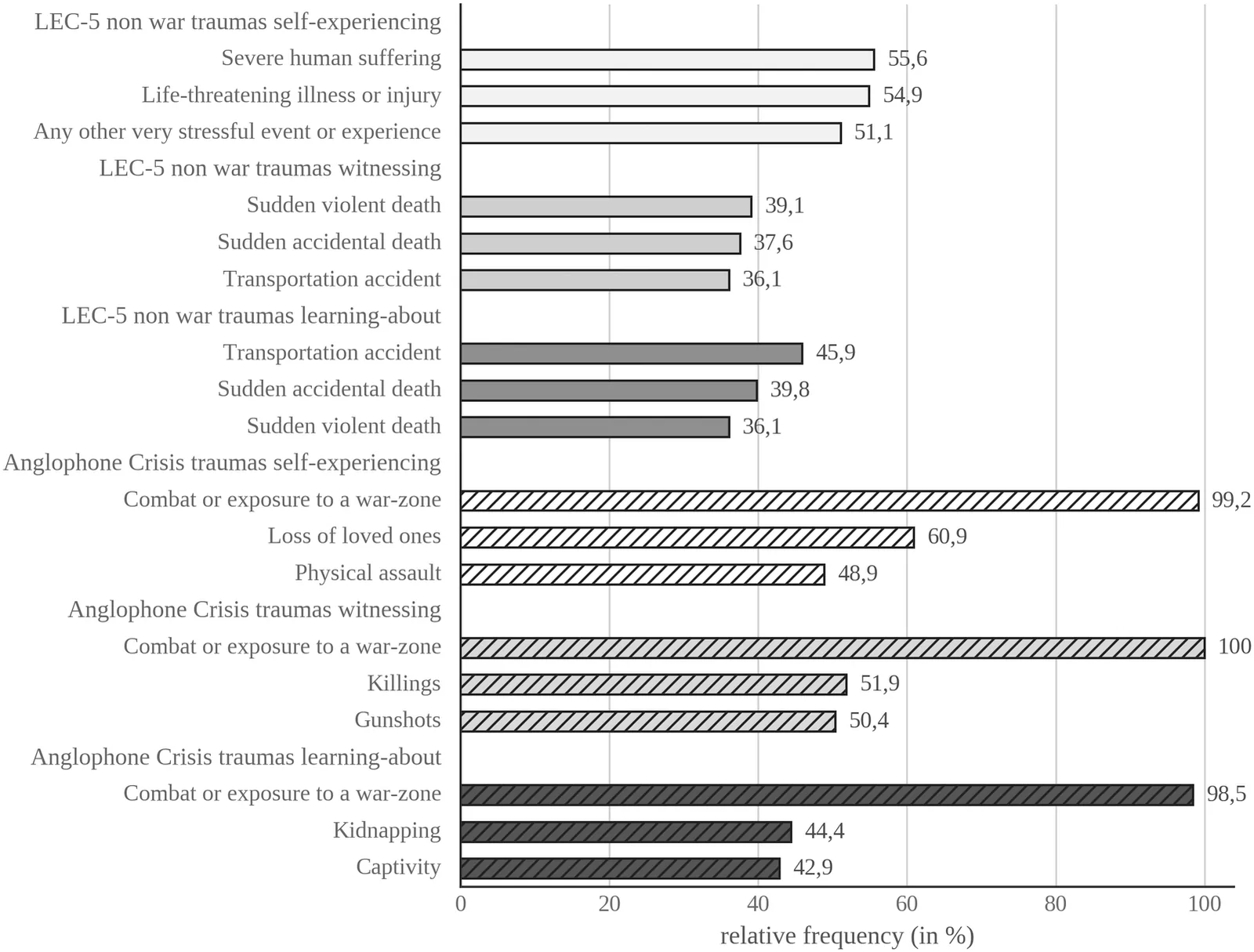
Top three frequencies of traumatic event types for the six categories of trauma-load scores. Values represent relative frequencies (%).

Figure 1 summarizes the three most frequently endorsed traumatic event types within each of the six trauma-load categories. The descriptive pattern indicates that Anglophone Crisis-related events were particularly frequent, especially in categories involving direct crisis exposure. In the self-experienced Anglophone Crisis category, detention or exposure in a war zone was endorsed by almost all participants, followed by loss of loved ones and other severe conflict-related events. In the witnessed and learned-about Anglophone Crisis categories, events such as killings and kidnapping were also highly prevalent.

The non-war-related LEC-5 categories showed a different profile. Frequently endorsed self-experienced non-war events included life-threatening illness or injury, whereas witnessed and learned-about non-war events prominently included sudden accidental death. This distinction supports the decision to analyze non-war-related and crisis-specific trauma loads separately. The descriptive findings show that the study population had experienced a broad range of potentially traumatic events, but the qualitative profile of exposure was strongly shaped by the Anglophone Crisis.

### Zero-order correlations

Table 2 presents means, standard deviations, and zero-order correlations among the main study variables. The mean PCL-5 total score was 34.68 (*SD* = 17.01), which is in the range often used to indicate clinically relevant PTSD symptom burden in screening contexts. The mean personal SoC score was 23.34 (*SD* = 12.95), and the mean SoCC score was 18.23 (*SD* = 8.58). Personal SoC and SoCC were not significantly correlated with each other, suggesting that the individual and community-level coherence constructs captured distinct perceptions in this displaced sample.

**Table 2:** Zero-order correlations, means, and standard deviations for the main study variables.

Variable	M	SD	Statistic	2	3	4	5	6	7	8	9
1. PCL-5 sum score	34.68	17.01	r	−.23	−.09	.17	.09	.03	.27	.20	−.05
			p	.007	.310	.056	.316	.727	.002	.022	.552
2. SOC sum score	23.34	12.95	r		.13	.16	−.04	.19	−.22	−.05	.09
			p		.149	.068	.682	.029	.009	.537	.297
3. SOCC sum score	18.23	8.58	r			−.01	−.11	.02	−.07	.05	.04
			p			.884	.219	.860	.413	.580	.660
4. LEC-5 non-war self-experienced	3	2	r				−.20	.02	.45	.24	.03
			p				.234	.857	<.001	.006	.772
5. LEC-5 non-war witnessed	3	2	r					.10	.13	.58	.05
			p					.273	.149	<.001	.556
6. LEC-5 non-war learned-about	3	3	r						−.09	<.01	.71
			p						.279	.960	<.001
7. Anglophone Crisis self-experienced	6	3	r							.24	−.13
			p							.006	.134
8. Anglophone Crisis witnessed	7	5	r								−.04
			p								.691
9. Anglophone Crisis learned-about	7	5	r								
			p								

PCL-5 = PTSD Checklist for DSM-5; SOC, Sense of Coherence; SOCC, Sense of Community Coherence. *r* = Pearson correlation coefficient; *p* = level of significance.

PCL-5 symptom severity was significantly negatively correlated with personal SoC (*r* = −0.23, *p* = .007), indicating that women with higher personal SoC tended to report lower PTSD symptom severity. In contrast, PCL-5 symptom severity was not significantly correlated with SoCC (*r* = –.09, *p* = .310). Among the trauma-load scores, PCL-5 symptom severity was significantly positively correlated with self-experienced Anglophone Crisis-related trauma load (*r* = .27, *p* = .002) and witnessed Anglophone Crisis-related trauma load (*r* = .20, *p* = .022). The correlations with non-war-related LEC-5 trauma-load scores were not significant. Personal SoC was negatively correlated with self-experienced Anglophone Crisis-related trauma load (*r* = –.22, *p* = .009), which is consistent with the possibility that higher direct trauma exposure is related to lower perceived coherence, although the cross-sectional design does not permit causal ordering.

### Prediction of PTSD symptom severity from trauma load

To identify the trauma-load category most strongly associated with PTSD symptom severity, the six trauma-load scores were entered into a multiple linear regression model predicting the PCL-5 sum score. The full model approached statistical significance, *F*(6,126) = 2.14, *p* = .053, adjusted *R^2^* = .05, and showed no problematic multicollinearity (maximum VIF = 2.02). After stepwise backward exclusion of non-significant predictors, the final model retained only self-experienced Anglophone Crisis-related traumatic event types as a significant predictor of PCL-5 symptom severity (Table 3).

**Table 3:** Regression model predicting PCL-5 symptom severity from the six trauma-load scores.

Predictor	Full model *β*	Full model p	Final model *β*	Final model p	Full 95% CI	Final 95% CI
4. LEC-5 non-war traumas—self-experienced	.04	.697			[−0.162, 0.242]	
5. LEC-5 non-war traumas—witnessed	−.03	.743			[−0.210, 0.150]	
6. LEC-5 non-war traumas—learned-about	.01	.943			[−0.264, 0.284]	
7. Anglophone Crisis trauma—self-experienced	.21	.029	.26	.002	[0.021, 0.399]	[0.095, 0.425]
8. Anglophone Crisis trauma—witnessed	.16	.144			[−0.055, 0.375]	
9. Anglophone Crisis trauma—learned-about	−.02	.842			[−0.218, 0.178]	

Predictor numbering corresponds to Table 2. *β* = standardized regression coefficient; *p* = level of significance. 95% CI = 95% confidence interval.

The final trauma-load model was significant, *F*(1,131) = 9.99, *p* = .002, adjusted *R*^2^ = .06. The standardized regression coefficient for self-experienced Anglophone Crisis-related trauma load was *β* = .26, *p* = .002. Thus, each increase in the number of self-experienced crisis-related traumatic event types was associated with higher PTSD symptom severity. Witnessed and learned-about crisis-related events, as well as the three non-war-related LEC-5 trauma-load indicators, did not remain significant predictors when analyzed in the full set of trauma-load variables. The corresponding 95% confidence intervals are reported in Table 3.

### Personal and community sense of coherence

The final step examined whether personal SoC and SoCC accounted for additional variance in PCL-5 symptom severity and whether they moderated the association between self-experienced Anglophone Crisis-related trauma load and PTSD symptoms. The full model included self-experienced crisis-related trauma load, personal SoC, SoCC, the interaction between trauma load and personal SoC, and the interaction between trauma load and SoCC. This model was statistically significant, *F*(5,127) = 3.17, *p* = .010, adjusted *R*^2^ = .05, and showed no evidence of problematic multicollinearity (maximum VIF = 1.24).

Neither interaction term was significant. The interaction between personal SoC and self-experienced Anglophone Crisis-related trauma load was not associated with PCL-5 symptom severity (*β* = –.08, *p* = .804), and the interaction between SoCC and trauma load was also non-significant (*β* = –.01, *p* = .899). These findings indicate that the association between trauma load and PTSD symptom severity was not statistically moderated by either personal or community coherence in the present sample.

After backward exclusion of non-significant predictors, the final model retained self-experienced Anglophone Crisis-related trauma load and personal SoC as significant predictors, *F*(2, 130) = 7.41, *p* < .001, adjusted *R*^2^ = .09, maximum VIF = 1.05 (Table 4). Higher self-experienced crisis-related trauma load predicted higher PCL-5 symptom severity (*β* = .23, *p* = .009), whereas higher personal SoC predicted lower PCL-5 symptom severity (*β* = –.18, *p* = .035). SoCC did not show a significant independent association and was removed from the final model. This pattern supports an interpretation of personal SoC as a protective correlate or resilience-related resource, but it does not support a claim of full mediation or moderation. The corresponding 95% confidence intervals are reported in Table 4.

**Table 4:** Regression model exploring personal sense of coherence, sense of community coherence, and interaction terms as predictors of PCL-5 symptom severity.

Predictor	Full model *β*	Full model p	Final model *β*	Final model p	Full 95% CI	Final 95% CI
2. SOC sum score	−.20	.028	−.18	.035	[−0.378, −0.022]	[−0.347, −0.013]
3. SOCC sum score	−.06	.517			[−0.241, 0.121]	
7. Anglophone Crisis trauma—self-experienced	.21	.016	.23	.009	[0.039, 0.381]	[0.057, 0.403]
SOC × Anglophone Crisis trauma—self-experienced	−.08	.804			[−0.712, 0.552]	
SOCC × Anglophone Crisis trauma—self-experienced	−.01	.899			[−0.164, 0.144]	

Predictor numbering corresponds to Table 2. *β* = standardized regression coefficient; *p* = level of significance. Interaction terms were calculated after mean-centering variables. 95% CI = 95% confidence interval.

## Discussion

This study examined trauma load, personal sense of coherence (SoC), and sense of community coherence (SoCC) as predictors of PTSD symptom severity among female internally displaced persons (IDPs) affected by the Anglophone Crisis in Cameroon. Three findings are central.

First, among six trauma-load indicators, self-experienced Anglophone Crisis-related traumatic event types were the only trauma-related predictor retained in the final regression model.

Second, personal SoC was inversely associated with PTSD symptom severity after adjustment for self-experienced crisis-related trauma.

Third, SoCC showed neither an independent association with PTSD symptom severity nor a moderating effect.

Together, the findings indicate that directly experienced conflict-related trauma and individual coherence resources were more closely related to PTSD symptoms than community coherence as measured in the present displacement context.

The prominence of self-experienced Anglophone Crisis-related trauma is consistent with the broader dose-response literature showing that cumulative traumatic exposure is associated with PTSD symptom severity in war-affected and displaced populations (5–7). The present results refine this pattern by showing that directly experienced crisis-related events, rather than non-war events, witnessed events, or learned-about events, carried the strongest independent association with PCL-5 symptom severity. This is clinically plausible because direct exposure often involves immediate threat to bodily integrity, helplessness, shame, or loss of control and may be strongly encoded in autobiographical memory (10, 11). The finding therefore highlights the importance of carefully assessing personally experienced conflict-related events when identifying women who may require trauma-focused support.

The absence of independent effects for witnessed and learned-about events should not be interpreted as evidence that these exposures are harmless. In a context of widespread political violence, witnessing violence or learning about violence affecting close others may contribute to a general climate of threat. However, such exposures may have been too common in the present sample to explain individual differences in PTSD symptom severity as strongly as directly experienced trauma. This distinction is practically relevant for low-resource humanitarian settings: brief assessment procedures should capture the full trauma profile, but intervention planning may need to prioritize the events that women experienced directly and that continue to drive re-experiencing, avoidance, and hyperarousal.

Personal SoC was inversely associated with PTSD symptom severity after controlling for self-experienced Anglophone Crisis-related trauma load. This finding is consistent with salutogenic theory and meta-analytic evidence linking stronger SoC to lower posttraumatic stress reactions (16, 18, 19). In displaced women, SoC may support recovery by strengthening the perception that current experiences can be understood, that internal and external resources are available, and that life remains meaningful despite trauma, loss, and uncertainty. Interventions that strengthen comprehensibility, manageability, and meaningfulness may therefore be valuable complements to trauma-focused care. Examples include psychoeducation, resource mapping, problem-solving support, narrative integration of traumatic memories, and meaning-oriented elements that help survivors reconnect traumatic experiences with a broader life story. Because of the cross-sectional design, these interpretations remain cautious: the data support personal SoC as a protective correlate or resilience-related resource, but they do not establish causal mediation. Reverse causality remains possible: more severe PTSD symptoms may themselves undermine perceived comprehensibility, manageability, and meaningfulness, rather than lower SoC only preceding PTSD symptoms.

The non-significant finding for SoCC is also important. Community coherence is theoretically relevant because communities can provide shared meaning, social support, collective efficacy, and practical resources (22, 24). Nevertheless, displacement may disrupt precisely those community processes. Women displaced from the North-West and South-West regions may be separated from communities of origin while simultaneously navigating unfamiliar host environments, humanitarian networks, religious groups, or temporary informal support systems. Under such conditions, community may no longer be a stable, bounded, and trusted source of coherence. The present findings therefore do not argue against community-based approaches; rather, they suggest that such approaches may first need to rebuild safety, trust, and access to support before community coherence can function as a resilience resource. Alternative explanations should also be considered. The SoCC scale may not have fully captured displacement-specific forms of community belonging, especially if participants differed in whether they were referring to their community of origin, the current host community, religious networks, humanitarian organizations, or informal support groups.

Clinically, the results support a combined model for women's mental health in humanitarian settings. Trauma-focused interventions remain essential because directly experienced crisis-related traumatic events were the strongest trauma-related predictor of PTSD symptom severity. Narrative Exposure Therapy and related approaches may be particularly relevant in contexts of repeated trauma because they aim to organize fragmented traumatic memories into a chronological autobiographical narrative (38). At the same time, care should strengthen individual coherence and coping without shifting responsibility for recovery onto survivors. For displaced women, personal coping is shaped by safety, poverty, housing instability, caregiving responsibilities, stigma, gender-based violence, and access to medical, legal, and psychosocial support. Interventions are therefore likely to be most useful when individual trauma processing is embedded within gender-sensitive and structurally informed humanitarian responses (1, 14).

Several strengths and limitations should be considered. The study focused on an underrepresented population of female IDPs in a Central African conflict context, differentiated non-war-related from Anglophone Crisis-related trauma, and distinguished self-experienced, witnessed, and learned-about exposure. It also examined personal and community coherence within the same model. At the same time, the cross-sectional design prevents causal conclusions; all measures were self-reported; and the sample consisted of women registered with two humanitarian organizations in Yaoundé, which may limit generalizability to unregistered IDPs, women living elsewhere, women remaining in conflict zones, or returnees. The sample size may also have limited power to detect small interaction effects, and the SoCC measure may not fully capture the complexity of community belonging after internal displacement. In particular, the cross-sectional design leaves open the possibility that PTSD symptoms influenced perceived SoC, and the null SoCC findings may partly reflect measurement limitations or ambiguity in the referent community after displacement.

Future research should examine trauma exposure, personal SoC, SoCC, and PTSD symptoms longitudinally and should include more detailed measures of social support, perceived safety, gender-based violence, caregiving burden, economic stress, and access to care. Mixed-methods studies could clarify how displaced women define community after displacement and how personal and collective forms of coherence develop over time. Intervention studies are needed to test whether trauma-focused care that explicitly strengthens comprehensibility, manageability, and meaningfulness can reduce PTSD symptom severity and improve functioning among internally displaced women.

## Conclusion

Among female IDPs affected by the Anglophone Crisis in Cameroon, self-experienced conflict-related trauma was the only trauma-load indicator retained as a predictor of PTSD symptom severity, while personal SoC was associated with lower symptom severity after adjustment for trauma load. SoCC did not show significant independent or moderating effects, possibly because displacement disrupts the social continuity required for community-level coherence to support resilience. The findings should not be interpreted as evidence of full mediation, but they do support the clinical relevance of individual coherence as a resilience-related resource. Trauma-focused and gender-sensitive interventions for displaced women should therefore strengthen personal coping and narrative integration while also addressing the social and structural disruption inherent in displacement.

## Data Availability

The datasets generated and analyzed for this study are not publicly available because they contain sensitive information from conflict-affected participants and because unrestricted sharing was not covered by the original consent procedures. De-identified data may be made available by the corresponding author upon reasonable request and subject to ethical and institutional approval.
